# Supplementary material for: Modeling glioblastoma heterogeneity as a dynamic network of cell states
Source: Mol Syst Biol. 2021 Sep 16;17(9):e10105. doi: 10.15252/msb.202010105 (PMC8444284; doi:10.15252/msb.202010105)
Supplement: Supplementary file 6 — Source Data for Figure 5 [file MSB-17-e10105-s004.zip › Figure5A_sourcedata/GSEA_3071/nosigdb_stateV.GseaPreranked.1622795159954/RICHARDS_VERHAAK_CANCERCELL_2010_MESENCHYMAL.html]

Details for gene set RICHARDS\_VERHAAK\_CANCERCELL\_2010\_MESENCHYMAL[GSEA]

|  || Dataset | state43071 |
| Phenotype | NoPhenotypeAvailable |
| Upregulated in class | na\_neg |
| GeneSet | RICHARDS\_VERHAAK\_CANCERCELL\_2010\_MESENCHYMAL |
| Enrichment Score (ES) | -0.653037 |
| Normalized Enrichment Score (NES) | -3.684021 |
| Nominal p-value | 0.0 |
| FDR q-value | 0.0 |
| FWER p-Value | 0.0 |
Table: GSEA Results Summary

  

Fig 1: Enrichment plot: RICHARDS\_VERHAAK\_CANCERCELL\_2010\_MESENCHYMAL      
 Profile of the Running ES Score & Positions of GeneSet Members on the Rank Ordered List

  

| PROBE | GENE SYMBOL | GENE\_TITLE | RANK IN GENE LIST | RANK METRIC SCORE | RUNNING ES | CORE ENRICHMENT || 1 | PDPN |  |  | 65 | 1.493 | 0.0054 | No |
| 2 | HEXA |  |  | 341 | 1.024 | -0.0353 | No |
| 3 | COL1A2 |  |  | 1028 | 0.614 | -0.1599 | No |
| 4 | EFEMP2 |  |  | 1300 | 0.532 | -0.2057 | No |
| 5 | TGOLN2 |  |  | 1714 | 0.434 | -0.2800 | No |
| 6 | UCP2 |  |  | 1779 | 0.422 | -0.2872 | No |
| 7 | WWTR1 |  |  | 1794 | 0.419 | -0.2849 | No |
| 8 | PLAU |  |  | 1863 | 0.408 | -0.2931 | No |
| 9 | ANXA1 |  |  | 2165 | 0.366 | -0.3466 | No |
| 10 | MRC2 |  |  | 2378 | 0.338 | -0.3834 | No |
| 11 | FNDC3B |  |  | 2617 | 0.308 | -0.4255 | No |
| 12 | COLGALT1 |  |  | 2623 | 0.307 | -0.4227 | No |
| 13 | SYPL1 |  |  | 2625 | 0.307 | -0.4193 | No |
| 14 | MAFB |  |  | 2898 | 0.279 | -0.4682 | No |
| 15 | SYNGR2 |  |  | 2933 | 0.276 | -0.4715 | No |
| 16 | FCGR2A |  |  | 2997 | 0.270 | -0.4804 | No |
| 17 | SWAP70 |  |  | 3329 | -0.271 | -0.5408 | No |
| 18 | LCP1 |  |  | 3470 | -0.294 | -0.5642 | No |
| 19 | CASP1 |  |  | 3648 | -0.323 | -0.5944 | No |
| 20 | TNFAIP3 |  |  | 3691 | -0.330 | -0.5985 | No |
| 21 | TRADD |  |  | 3696 | -0.331 | -0.5954 | No |
| 22 | VDR |  |  | 3793 | -0.348 | -0.6097 | No |
| 23 | PHF11 |  |  | 3819 | -0.353 | -0.6102 | No |
| 24 | LY96 |  |  | 3829 | -0.354 | -0.6077 | No |
| 25 | FURIN |  |  | 3861 | -0.361 | -0.6094 | No |
| 26 | CPQ |  |  | 3933 | -0.374 | -0.6185 | No |
| 27 | ITGB2 |  |  | 3948 | -0.377 | -0.6167 | No |
| 28 | YAP1 |  |  | 4041 | -0.398 | -0.6297 | No |
| 29 | EHD2 |  |  | 4051 | -0.399 | -0.6266 | No |
| 30 | LAMB1 |  |  | 4115 | -0.413 | -0.6338 | No |
| 31 | TRIM22 |  |  | 4138 | -0.419 | -0.6330 | No |
| 32 | PLBD1 |  |  | 4225 | -0.448 | -0.6442 | No |
| 33 | RAB32 |  |  | 4251 | -0.457 | -0.6435 | No |
| 34 | TRIM38 |  |  | 4264 | -0.461 | -0.6403 | No |
| 35 | ICAM3 |  |  | 4269 | -0.463 | -0.6356 | No |
| 36 | COL8A2 |  |  | 4277 | -0.467 | -0.6313 | No |
| 37 | LTBP1 |  |  | 4322 | -0.477 | -0.6341 | No |
| 38 | ASL |  |  | 4369 | -0.491 | -0.6370 | No |
| 39 | BLVRB |  |  | 4371 | -0.491 | -0.6313 | No |
| 40 | TMBIM1 |  |  | 4427 | -0.512 | -0.6358 | No |
| 41 | FHL2 |  |  | 4451 | -0.521 | -0.6340 | No |
| 42 | CASP4 |  |  | 4551 | -0.565 | -0.6463 | Yes |
| 43 | PYGL |  |  | 4554 | -0.567 | -0.6399 | Yes |
| 44 | RAB11FIP1 |  |  | 4577 | -0.575 | -0.6372 | Yes |
| 45 | ITGA5 |  |  | 4580 | -0.576 | -0.6307 | Yes |
| 46 | SCPEP1 |  |  | 4589 | -0.579 | -0.6253 | Yes |
| 47 | PIGP |  |  | 4605 | -0.591 | -0.6211 | Yes |
| 48 | MAN1A1 |  |  | 4640 | -0.614 | -0.6203 | Yes |
| 49 | RUNX2 |  |  | 4697 | -0.643 | -0.6234 | Yes |
| 50 | POLD4 |  |  | 4705 | -0.646 | -0.6170 | Yes |
| 51 | RRAS |  |  | 4717 | -0.657 | -0.6113 | Yes |
| 52 | MVP |  |  | 4760 | -0.686 | -0.6111 | Yes |
| 53 | PLS3 |  |  | 4771 | -0.692 | -0.6048 | Yes |
| 54 | LGALS1 |  |  | 4784 | -0.699 | -0.5987 | Yes |
| 55 | S100A13 |  |  | 4791 | -0.705 | -0.5914 | Yes |
| 56 | IGFBP6 |  |  | 4800 | -0.715 | -0.5844 | Yes |
| 57 | ARPC1B |  |  | 4818 | -0.730 | -0.5789 | Yes |
| 58 | BNC2 |  |  | 4819 | -0.730 | -0.5702 | Yes |
| 59 | CNN2 |  |  | 4840 | -0.749 | -0.5651 | Yes |
| 60 | NPC2 |  |  | 4841 | -0.750 | -0.5561 | Yes |
| 61 | CLIC1 |  |  | 4899 | -0.809 | -0.5574 | Yes |
| 62 | DSE |  |  | 4903 | -0.818 | -0.5482 | Yes |
| 63 | SLC16A3 |  |  | 4949 | -0.863 | -0.5465 | Yes |
| 64 | SEC24D |  |  | 4953 | -0.871 | -0.5366 | Yes |
| 65 | CTSB |  |  | 5007 | -0.948 | -0.5355 | Yes |
| 66 | TNFAIP8 |  |  | 5009 | -0.949 | -0.5243 | Yes |
| 67 | LGALS3 |  |  | 5039 | -1.017 | -0.5177 | Yes |
| 68 | LRRFIP1 |  |  | 5044 | -1.025 | -0.5062 | Yes |
| 69 | ANXA2 |  |  | 5055 | -1.036 | -0.4958 | Yes |
| 70 | COPZ2 |  |  | 5069 | -1.073 | -0.4854 | Yes |
| 71 | CEBPB |  |  | 5076 | -1.088 | -0.4735 | Yes |
| 72 | UAP1 |  |  | 5082 | -1.100 | -0.4613 | Yes |
| 73 | EMP3 |  |  | 5084 | -1.113 | -0.4482 | Yes |
| 74 | MYH9 |  |  | 5090 | -1.125 | -0.4357 | Yes |
| 75 | LOX |  |  | 5092 | -1.128 | -0.4224 | Yes |
| 76 | SHC1 |  |  | 5094 | -1.133 | -0.4090 | Yes |
| 77 | PROCR |  |  | 5097 | -1.139 | -0.3958 | Yes |
| 78 | ANXA4 |  |  | 5106 | -1.172 | -0.3833 | Yes |
| 79 | SRPX2 |  |  | 5108 | -1.176 | -0.3694 | Yes |
| 80 | SP100 |  |  | 5126 | -1.230 | -0.3580 | Yes |
| 81 | RBMS1 |  |  | 5129 | -1.240 | -0.3435 | Yes |
| 82 | TIMP1 |  |  | 5134 | -1.251 | -0.3293 | Yes |
| 83 | TGFBI |  |  | 5136 | -1.259 | -0.3144 | Yes |
| 84 | CAST |  |  | 5142 | -1.277 | -0.3001 | Yes |
| 85 | SERPINE1 |  |  | 5161 | -1.377 | -0.2871 | Yes |
| 86 | P4HA2 |  |  | 5164 | -1.393 | -0.2708 | Yes |
| 87 | PLAUR |  |  | 5185 | -1.479 | -0.2569 | Yes |
| 88 | FXYD5 |  |  | 5195 | -1.527 | -0.2404 | Yes |
| 89 | S100A11 |  |  | 5196 | -1.536 | -0.2220 | Yes |
| 90 | THBS1 |  |  | 5197 | -1.538 | -0.2036 | Yes |
| 91 | MYOF |  |  | 5207 | -1.590 | -0.1863 | Yes |
| 92 | NRP1 |  |  | 5211 | -1.609 | -0.1676 | Yes |
| 93 | S100A4 |  |  | 5238 | -1.817 | -0.1509 | Yes |
| 94 | CLEC2B |  |  | 5255 | -2.049 | -0.1294 | Yes |
| 95 | DAB2 |  |  | 5261 | -2.217 | -0.1038 | Yes |
| 96 | COL5A1 |  |  | 5274 | -2.455 | -0.0768 | Yes |
| 97 | ARHGAP29 |  |  | 5278 | -2.576 | -0.0465 | Yes |
| 98 | COL1A1 |  |  | 5294 | -4.144 | 0.0002 | Yes |
Table: GSEA details [plain text format]

  

Fig 2: RICHARDS\_VERHAAK\_CANCERCELL\_2010\_MESENCHYMAL: Random ES distribution      
 Gene set null distribution of ES for **RICHARDS\_VERHAAK\_CANCERCELL\_2010\_MESENCHYMAL**
